# 803. Plasma GDF15 Is Persistently Elevated in Mice and Humans After Burn Injury

**DOI:** 10.1093/jbcr/irag033.181

**Published:** 2026-04-06

**Authors:** Thomas O Vogler, Mara R Evans, Kevin M Najarro, Rachel H McMahan, Alexandra Halevi, Arek J Wiktor, Elizabeth J Kovacs

**Affiliations:** University of Colorado Anschutz, Aurora, CO; University of Colorado Anschutz, Aurora, CO; University of Colorado Anschutz, Aurora, CO; University of Colorado Anschutz, Aurora, CO; UCHealth Burn and Frostbite Center - Anschutz Medical Campus, Aurora, CO; UCHealth Burn and Frostbite Center - Anschutz Medical Campus, Aurora, CO; University of Colorado Anschutz, Aurora, CO

## Abstract

**Introduction:**

Growth differentiation factor 15 (GDF15) is a cytokine in the TGFβ superfamily whose expression is increased in response to cellular stress. In disease states such as cancer cachexia and cardiovascular disease, GDF15 acts both centrally and peripherally to mediate appetite, body mass, and skeletal muscle energy expenditure. Recent evidence shows elevated plasma GDF15 levels immediately after a burn injury in humans, however, if elevated GDF15 levels persist and what effects this has on the hypermetabolic burn response is unknown.

**Methods:**

Patients with greater than 20% total body surface area (TBSA) thermal burns admitted to our ABA-verified burn center during June–September 2025 were included in the study. Ten patients met inclusion criteria (average age 39.4; 80% male; average TBSA 27.25%). GDF15 plasma levels were tracked on admission and then weekly until discharge. GDF15 levels were compared to the normal range < 750 pg/mL. In mice studies, ten- to twelve-week-old male C57B/6 J mice were given a 12% TBSA scald burn. Total body weights were measured daily. Plasma GDF15 levels and muscle weights were measured at the time of euthanasia. GDF15 levels were compared to sham-injured control mice.

**Results:**

In humans with greater than 20% TBSA burns, plasma GDF15 levels were elevated on admission and then increased over the next week (994 ± 185 vs 1819 ± 130 pg/mL; *p*=.003). At three weeks post injury, 100% of patients still admitted had GDF15 levels above the normal range with an average of 1429 ± 253 pg/mL. In mice, GDF15 plasma were increased at 24 hrs (1633 ± 365 vs 96 ± 9 pg/ml; *p*=.0006) and remain elevated for one week (274 ± 110 pg/ml; *p*=.03). In mice, high GDF15 levels correlated with total weight loss and muscle mass loss during the first week. By two weeks, GDF15 levels returned to near baseline, which correlated with improving muscle mass and total weight in mice.

**Conclusions:**

Plasma GDF15 increased immediately post burn injury and remained high in mice and humans. This is the first study to show GDF15 levels are persistently elevated in mice and humans after burn injury. In mice, the rise and fall of GDF15 levels are inversely correlated with muscle and total body weights. Given the role of GDF15 in appetite suppression and skeletal muscle energy expenditure, future studies are needed to elucidate the effects that the rise in GDF15 levels has on hypophagia and hypermetabolism following burn injuries.

**Applicability of Research to Practice:**

GDF15 is a centrally acting cytokine that is increased in patients with burn injuries. Persistent elevation of GDF15 in patients with burns could contribute to the prolonged hypermetabolic response seen with burn injury. More animal studies are needed to test causality and to elucidate the mechanism of action of GDF15, however GDF15 remains an intriguing potential therapeutic target in patients with large TBSA burns.

**Funding for the study:**

NIH.

